# Understanding processes of risk and protection that shape the sexual and reproductive health of young women affected by conflict: the price of protection

**DOI:** 10.1186/s13031-017-0117-x

**Published:** 2017-08-17

**Authors:** Aisha Hutchinson, Philippa Waterhouse, Jane March-McDonald, Sarah Neal, Roger Ingham

**Affiliations:** 1 Institute for Applied Social Research, University of Bedfordshire, Luton, LU1 3JU Bedfordhire UK; 2 Centre for Social Development in Africa (CSDA), University of Johannesburg, Johannesburg, South Africa; 3 Faculty of Wellbeing, Education and Language Studies, The Open University, Milton Keynes, SO17 1BJ UK; 4 Health Sciences, University of Southampton, Hampshire, SO17 1BJ UK; 5 Department of Social Statistics and Demography, University of Southampton, Hampshire, SO17 1BJ UK; 6 Centre for Sexual Health Research, Psychology, University of Southampton, Southampton, SO17 1BJ UK

**Keywords:** Sexual and reproductive health, Risk, Protection, Resilience, Gender, Young women, Ecological framework, Post-conflict care

## Abstract

**Background:**

It is assumed that knowing what puts young women at risk of poor sexual health outcomes and, in turn, what protects them against these outcomes, will enable greater targeted protection as well as help in designing more effective programmes. Accordingly, efforts have been directed towards mapping risk and protective factors onto general ecological frameworks, but these currently do not take into account the context of modern armed conflict. A literature overview approach was used to identify SRH related risk and protective factors specifically for young women affected by modern armed conflict.

**Processes of risk and protection:**

A range of keywords were used to identify academic articles which explored the sexual and reproductive health needs of young women affected by modern armed conflict. Selected articles were read to identify risk and protective factors in relation to sexual and reproductive health. While no articles explicitly identified ‘risk’ or ‘protective’ factors, we were able to extrapolate these through a thorough engagement with the text. However, we found that it was difficult to identify factors as either ‘risky’ or ‘protective’, with many having the capacity to be both risky and protective (i.e. refugee camps or family). Therefore, using an ecological model, six environments that impact upon young women’s lives in contexts of modern armed conflict are used to illustrate the dynamic and complex operation of risk and protection – highlighting processes of protection and the ‘trade-offs’ between risks.

**Conclusion:**

We conclude that there are no simple formulaic risk/protection patterns to be applied in every conflict and post-conflict context. Instead, there needs to be greater recognition of the ‘processes’ of protection, including the role of ‘trade-offs’ (what we term as ‘protection at a price’), in order to further effective policy and practical responses to improve sexual and reproductive health outcomes during or following armed conflict. Focus on specific ‘factors’ (such as ‘female headed household’) takes attention away from the processes through which factors manifest themselves and which often determine whether the factor will later be considered ‘risk inducing’ or protective.

## Background

Blum and Mmari (2005) propose a conceptual framework which locates risk and protective factors associated with the sexual and reproductive health (SRH) of young people in developing countries in an ecological model [1]. This assumes that knowing what factors are likely to increase poor SRH outcomes for young women (including early first sex or early first birth), and how they operate, will help target young women at risk of negative health outcomes and help to design more effective programmes. The framework views young people as living in multiple milieus (macro/institutional, community, school, family, peers and individual), each of which may be a source of both risk and protection. This model is presented as functioning across developing contexts. Predominately, work on SRH has taken a population approach, with limited consideration of its suitability for application to specific contexts, such as conflict. However, a small body of emerging evidence suggests that processes of risk and protection are, to a large extent, contextually determined and need to be understood in relation to distinct groups of people in specific contexts [2–4]. While there has been some exploration of specific risk and protective factors in relation to conflict and the short or long term impacts upon some aspects of health [5–7], based on this literature overview, there appears to be little that has been specific to SRH and young women.

The inter-agency field manual on reproductive health in humanitarian settings highlights the importance of identifying protective factors within initial assessments; however, little is known as to how conflict may undermine protective processes or increase the ‘trade-off’ between risks that may be undertaken to increase protection [8]. Conflict is likely to have a powerful impact on the ecology of young people and an ecological model enables a more comprehensive exploration of protective processes, with socio-cultural contexts becoming the focus of attention rather than individual attributes [7].

Risk and risk factors are often used and understood as notions of statistical risk, and commonly associated with increasing the likelihoods of negative outcomes or problem behaviours. Protective factors operate in the context of risk and may be understood as the resources that support and assist an individual, family or community to manage, restrict and/ or overcome difficulties and adversity, and reduce risk [9]. Such conceptualisations of risk and protective factors suggest that they are static and generalisable factors. However, the dynamic nature of a factor, and whether or how it serves to protect or increase risk, can only be understood when wider processes of risk and protection are identified (i.e. how it came to be that certain choices were made or that a particular context occurred). This may involve several dynamic factors, as well as combinations of risk and protective factors (or a trade-off between them), each of which can produce different outcomes for any individual.

Adopting this position, and building upon Blum and Mmari’s (2005) ecological framework [1], an overview of the risk and protective factors highlighted by the literature on the young women’s SRH in conflict is presented. Yet rather than describing a neat set of risk and protective factors that can be used to underpin policy and practice responses, we present the complex processes of protection that often dictate whether a context or choice is risk inducing or protective. Through this work, we argue that we need to better understand and pay more attention to these processes, the trade-offs which occur and the price often paid by young women through it.

## Methods

This paper adopts a literature overview approach (Grant and Booth 2009), providing a narrative of the relevant literature [10]. A search of the literature was performed using Web of Knowledge, limited to the title and abstract. The search period was from 2000 to 2013, and only papers in English were included. The search was driven by the overarching question ‘What are the risk and protective factors associated with the sexual and reproductive health of young women in contexts of armed conflict?’ The search strategy combined terms according to four broad themes:

- Conflict: conflict OR conflict affected OR fragile states OR war OR trauma OR violence
- Age: child* OR youth OR adolescen* OR teen OR young person*
- Conflict affected countries: Afghanistan OR Angola OR Burundi OR Central African Republic OR Chad OR DRC OR Congo OR Cote d’Ivoire OR Eritrea OR Guinea OR Guinea-Bissau OR Liberia OR Mali OR Myanmar OR Burma OR Nepal OR Sierra Leone OR Sri Lanka OR Somalia OR Sudan OR South Sudan OR Timer-Leste OR Togo OR West Bank OR Gaza OR Iraq OR Libya OR Syria
- Sexual and reproductive health: sexual and reproductive health OR childbearing OR pregnancy OR sexual activity OR early marriage

The search also involved hand searches of relevant journals, and the following up of citations, appropriate grey literature and key authors. Different types of literature (quantitative, qualitative, conceptual and discussion pieces, and grey literature) were included. The initial inclusion criteria focused on literature that explicatively discussed conflict, and sexual and reproductive health in relation to this. In addition, we restricted our focus to literature concerned with females and included those of any age between 8 and 18 years. Where age ranges were not clarified, articles that made references to adolescents, girls or youth were retained.

**Fig. 1:**
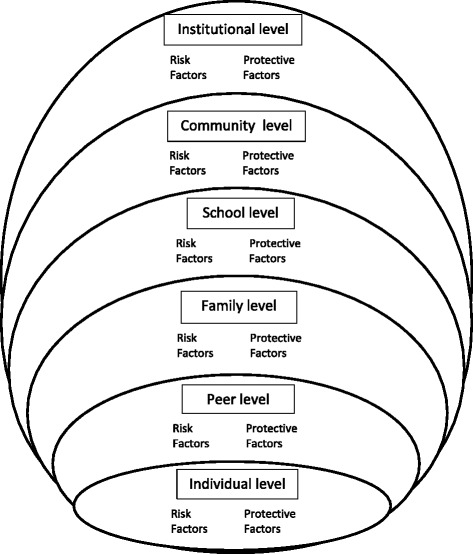
Risk and protective factors mapped onto an ecological framework

Identified literature was read to ascertain risk and protective factors associated with various poor sexual and reproductive health care outcomes, such as unmet need for contraception, early age at first birth, maternal mortality, infant mortality and sexually transmitted diseases. No explicit appraisal of the methodological quality of each piece was undertaken. Whilst we found little research that explicitly identified ‘risk’ and ‘protective’ factors, these were extrapolated by a thorough reading of the text. However, we found that these extrapolated factors were not clear cut, and many factors were found to be both protective and risky; for example, refugee camps offer protection against some poor sexual health outcomes through access to services but they can also be risky environments for SRH, especially in relation to sexual violence. An ecological framework of risk and protective factors, based on the pre-populated model used by Blum and Mmari (2005) (Figure 1) was used to analyse the literature thematically and present the results [1]. Identified factors were mapped according to six environments that were also used by Blum and Mmari; macro/institutional; Community; school; family; peer and individual. In a second stage, further literature was identified to populate certain levels of the framework, which were under-represented in the literature accessed thus far. For the macro/institutional level, for example, we draw on literature that related to sexual and reproductive health in conflict more generally, and the consequences of conflict on health systems where these could also impact on young women. Literature that describes the impact of conflict on education was used at the school level. Finally, literature concerned with the consequences of conflict on peer relationships and the civic participation of young people was included at the peer level.

## Processes of risk and protection

### Macro/institutional level environment

#### Unstable governance

Armed conflict can dramatically change the way young women access and benefit from (in theory, at least) structures such as legislative justice mechanisms, stable governance and policing which protect them from sexual violence or coercion, as well as processes for participation and demonstration which allow young women to voice their concerns. Issues of insecurity and fear of reprisal and attack can therefore limit access to health services [11–13]. Similarly, progressive social policy for SRH, which facilitates sexual education and access to family planning methods can be curtailed as well as livelihood safety nets to prevent destitution.

#### Poor infrastructure

Important health and education infrastructures that facilitate access to good quality SRH services can be severely affected during times of conflict. Verley (2010) reports, how during Shia-Sunni hostilities in Gilgit Town, Pakistan, obstetric service access and provision were severely reduced following the targeting, killing and exclusion of particular faith-based groups in hospitals and clinics, resulting in increased maternal morbidity and mortality [14]. During the Rwandan genocide an estimated 80% of health professionals were killed or fled the country, and medical supplies and equipment were heavily looted and destroyed [15]. More recently, attacks on professionals has caused a deficiency of healthcare providers in Iraq as many have left the country causing disruption to services (Mowafi 2010) [16]. Similarly, during the civil war in Mozambique, Renamo specifically targeted health and education facilitates in an effort to destabilise the country [17].

#### Institutional settings

The institutional setting of SRH services can change – as opposed to disappearing altogether - during conflict. For some young women, there is better access to SRH in refugee camps, or other displaced contexts, compared to their usual home [18]. The Reproductive Health Response in Conflict Consortium and the Inter-agency Working Group on Reproductive Health in Crisis, for example, have spent recent years increasing the priority of reproductive health in crises and have developed a wide range of responses for organisations responding to humanitarian disasters [8]. Nonetheless, despite humanitarian efforts, young people’s SRH can be neglected at the institutional level in contexts of displacement. Abdelmoneium (2010) and Wayte et al. (2008) both found that a focus on safe motherhood in Sudan and Timor-Leste, respectively, resulted in other aspects of SRH being side-lined because of limited resources and services, and the prioritisation of life-saving and emergency services [19, 20].

New institutional settings like refugee camps can also increase the risk of Sexual and Gender Based Violence (SGBV); Stavrou’s (2004) fieldwork in Angola identified the location of Internally Displaced Persons (IDP) camps close to military encampments as a contributory factor for the harassment faced by females [21]. Threats to sexual safety can also come from within camps as a result of the breakdown of social norms and deficient security [22, 23], and perpetrators can include humanitarian staff. Fear of sexual attacks and harassment can place restrictions on female mobility and, in the example of displaced Syrian women and girls, there are reports of greater limits to their freedom and mobility in their host countries with concern about attacks being the greatest for those unmarried [24].

#### Livelihoods

Due to the impact of conflict on the macro-economic context, conflict can also have profound changes on the livelihood strategies of households, which can put young women at risk of poor SRH outcomes. In rural Nepal, displacement caused by Maoist and threats from security forces caused a disruption and loss to agricultural livelihoods [25]. As a result, a substantial proportion of conflict-affected girls reported themselves to be working in contexts (for example, hotels and wine shops) where they feared sexual abuse and exploitation. Conflict in Northern Uganda and Southern Sudan also resulted in the engagement of young women in transactional sex as a means of family survival where access to farming was restricted [26]. It should be noted that, in relatively rare circumstances, the engagement of women in new livelihood strategies - such as trading - can support a sense of empowerment and autonomy due to their increased economic importance in the household, although this empowerment is rarely translated into greater representation at the community level [26].

At the Macro level, evidence highlights the ways in which SRH services are undermined. However, institutional adaptation to refocus and prioritise SRH provision within refugee camps and displacement centres has resulted in improved SRH access for some women, though such gains can occur in the context of increased sexual threat.

### Community level environment

*Each community has its own norms, beliefs and attitudes that determine how much autonomy and mobility a girl has, how easily she is able to enjoy and exercise her rights, whether she is safe from violence, whether she is forced into marriage, how likely she is to become pregnant, or whether she can resume her education after having had a child.* (United Nations Population Fund (UNFPA), 2013, p36) [27].

The extract above describes the complex, and often contradictory, impact of cultural norms and values on the SRH of young women. During times of conflict the breakdown of social cohesion and norms in a society can increase the risk to young women of negative sexual outcomes [28], particularly when protective mechanisms located in family and community structures are disrupted. The normalisation of sexual violence, such as rape, is reflected in the identification of perpetuators as civilians and its continuation into the post-conflict period [29]. Kalisya et al.’s (2011) analysis of HEAL Africa’s hospital records in the Democratic Republic of Congo (DRC) between the period 2006–2008 (post-conflict), found that in the majority of sexual assaults of presenting child victims the offender was a civilian, and in 74% of cases was known to the victim [30]. A similar pattern was found for child survivors presenting themselves to the Panzi Hospital in Eastern DRC [31].

#### Community level protection

When considering the community environment as a sphere of influence in relation to sexual violence, War Child (2013) suggests that local structures are at the centre of solutions to protect young women from sexual violence and there are examples of communities coming together to provide protection for young people [29]. Kottegoda et al. (2008), for example, drew attention to the protective nature of traditional midwives in contexts of conflict when access to formal medical access and support was reduced [32]. Footer et al. (2014) found that health workers, community/village leaders and local health organisations in Eastern Burma were active in devising strategies to maintain the provision of health services, despite attacks [13]. Communities have also been key in ensuring the continuity of education - which is widely considered as a key protective factor for young women. In Afghanistan, trusted female members of the community provided home schooling to girls during the Taliban’s ban on female education [33]. Community action can also be vital in providing places of security and sources of support for children separated from their families. In the case of the night commuters in Northern Uganda, local volunteers with the Peace Foundation Charity helped secure safe sleeping arrangements for young women, also providing supervision and guidance [34].

#### Changing norms

Conflict, through the breakdown of traditional norms, has the potential to challenge or change harmful practices [8]. Rajasingham-Senanayake (2004), for example, observed that challenged gender norms due to females’ roles as armed combatants, income generators and household heads during conflict in Sri Lanka, resulted in the increased agency of women which continued in the post-conflict period [35]. By contrast, changes in sexual behaviours during times of conflict can set young women on a track of high risk behaviours that continue into peacetime [28]. Transactional sex, for example, which may be instigated for survival during war, might continue to be used for material provision in peacetime, either due to lack of options, or to supplement other ways of securing income. Conflict can change norms of what is acceptable and what is a priority, although these changes may or may not be sustained in post-conflict times. Burman and McKay (2007), for example, note the marginalisation of young mothers after conflict (even when as a result of forced marriage) [36], as community members can become highly protective of gender and gender roles following conflict [8].

At the community level, both risk and protection can be seen to operate in different ways to either promote or undermine SRH outcomes for young women.

### School level environment

#### Access to education

Accessing education, mainly through schools, has been identified as a key determinant and protective factor in relation to most measures of SRH in developing contexts [1, 27]. However, it is well documented that conflict can significantly affect the school environment [29]. In 2011, 20 million out-of-school young people, roughly equal gender split, were living in countries affected by conflict [37]. In qualitative fieldwork conducted in Angola and Sierra Leone, girls discussed how their involvement with armed groups stopped them being able to attend school, with few having returned in the aftermath of war [21, 38]. Indirect interruption to education caused by damage to school infrastructure and possible loss of professional life as a result of conflict, has also been recorded in countries such as Mozambique, East Timor, Afghanistan and Sierra Leone [22, 39, 40]. Whilst several international conventions and resolutions stipulate the right of children to education, with no exceptions for periods of conflict and post-conflict, the tendency to focus on primary education can entail a relative lack of attention being paid to secondary schools in these settings [39].

In contexts of insecurity, the school environment can actually place additional risks on young women’s SRH. In Mozambique, Northern Uganda and Burundi, schools have been sites of abduction by rebel and government forces [41, 42]. In Sri Lanka, rebel groups carried out recruitment activities in nearby schools with the aim of persuading ‘voluntary’ enrolment [42]. In 2004, in Beslan, Russia, 1300 children and adults were taken hostage during the school day resulting in the death of 329 - including 189 students [7]. Young women can also be placed at increased risk of sexual violence or abuse on their journey to and from school, and sometimes from the very professionals who are meant to protect them. In West African refugee camps, teachers have been reported to bribe students with the promise of good grades in exchange for sexual favours, [43] although this is not unique to such contexts.

In rare circumstances, however, access to a safe school environment can improve in conflict. United Nations High Commissioner for Refugees (UNHCR)-supported education of Liberian children and young people in Guinean refugee camps was reported to be better quality than the education that was received in Liberia in the period prior to the conflict (1980–1989) [44]. The protective role of education in conflict is also reflected by the inclusion of education in United Nations (UN) resolutions designed to ensure the security of children in contexts of armed conflict [42]. Despite recognition that education should continue to serve as a protective factor during conflict for promoting SRH outcomes, evidence shines a light on how it may also increase young women’s sexual risk.

### Family level environment

#### Parents

Parental figures play an important role in the transition from childhood to adulthood, including in relation to SRH. Across the global south, Blum and Mmari (2005) found that living with both parents and family stability/connection was a protective factor in relation to early sexual debut, conception and childbearing [1]. As role models, parents, for example, can on the one hand, instil the importance of gender equality between men and women in relation to decision-making or, on the other hand, perpetuate the dominance of men in social relationships resulting in unequal power relationships [27]. Family structures may help young women develop negotiation skills and encourage them to make their own decisions regarding life choices, including condom use.

#### Family structures

Conflict can increase the protective role of families as young women tend to be at higher risk of rape, sexual exploitation and abuse when cut off from family structures [45]. In Angola and Sierra Leone, former girl soldiers frequently describe how their abductions were simultaneously accompanied by orphanhood when their parents were killed during village raids [21, 38, 41]. In the context of refugee camps, young women without families are the most vulnerable to sexual exploitation in exchange for monetary and material goods, including aid [43]. Practical logistics - such as where sanitation or cooking facilities are located - all have implications for sexual safety of young women [46]. However, in Northern Uganda, families actually used separation as a strategy to protect children from negative sexual experiences. Young ‘night commuters’ are sent from IDP camps to spend nights in nearby towns to reduce the risk of sexual violence and abduction by rebel groups [47]. Nonetheless, the insecurity of young women in mobility, commuters’ sites and public places at night - combined with non-gendered segregated sleeping and a lack of adult supervision – means that girls still experience sexual harassment and abuse, including from male night commuters [48].

#### Role of families

In contexts where accessing formal education or health services is impossible or dangerous, conflict heightens the protective capacity of families as sources of information and providers of care. Families support access to care when young women are giving birth through traditional birth attendants (who can play an important supportive role) especially when all other forms of formal health care are inaccessible or have been destroyed [46]. Nonetheless, reliance on the family regarding sexual and reproductive health knowledge can increase the risk of misleading, inaccurate or incomplete information [27].

#### Household vulnerabilities

Conflict can take its toll on the protective nature of family structures through changing roles within the household. Absence of males from the household, through conflict mortality, imprisonment and military membership, can leave households vulnerable to poverty and result in the engagement of females in economic activities which increase the risk of poor SRH [49]. Young women may feel compelled to marry early or take on economic activities which put them at increased risk of SGBV, or engage in transactional sex to provide for their family when there are limited options for securing livelihoods through other means [25, 27]. In the context of conflict, the avoidance of death, starvation and destitution is likely to be prioritised above the long term consequences of early motherhood. Conflict can also influence the interaction and relationships between family members; Catani et al. (2008) propose the idea that ‘cycles of violence’ do not just apply to the intergenerational context, but also to the transfer of behaviour from war to family violence. In their sample of Tamil youth, linear regression analysis revealed that previous exposure to war, measured by the number of events, was a significant predictor of the experience of family violence [50].

#### Early marriage

Whilst early and forced marriages certainly occur outside conflict affected regions, the literature we consulted reveals that such marriages within conflict affected regions have additional dimensions and complexities. Families often believe that marriage can provide security against the risk of SGBV during conflict, for example. Kottegoda et al. (2008), using semi-structured interviews, found that early marriage was described as a protective strategy used by families to reduce the risk of daughters being ‘recruited’ or abducted into military factions [32]. Similarly, Swaine and Feeny (2004) found early marriage was used as a strategy by families to protect girls from violence in Kosovo [51]. In Angola, married young women were actually reported to be less likely to be abducted during raids on villages [21]. Furthermore, amongst SGBV victims presenting at Panzi hospital in Eastern DRC, women and girls who were single without ever being married were six times more likely to be held captive for the purpose of sexual violence for more than 24 h in comparison to those married, abandoned or widowed [52]. Marriage can also be used by families as a form of justice to protect the honour of girls in the occurrence of SGBV [31, 32].

Anecdotal reports suggest that early marriages are increasing in Syrian families, and occurring at a younger age, as a result of conflict factors such as increased family poverty, female withdrawal from education due to barriers imposed by armed conflict and displacement, and increased risk of sexual violence of unmarried adolescents [24, 53]. Nonetheless, it has been noted that the changing nature of early marriage, driven by the conflict in Syria, can increase risk of sexual abuse for women as economic and social ties are broken between families, and marriages are arranged outside long established social networks, and without official marriage contracts [53]. Thus, evidence shows that early and arranged marriages may increase and/or decrease negative SRH outcomes in contexts of conflict.

The role of parents, families and family structure is evidenced to play a significant role in managing, reducing or exposing young women to increased SRH risk, highlighting the complex and often contradictory nature of risk and protective factors and processes in conflict contexts.

### Peer level environment

#### Peers

As individuals enter young adulthood, peers become an increasing influence, especially in relation to SRH [7]. While peers can create a negative culture and encourage risky sexual behaviours, they can also be a force for good. However, there seems to be little reflection of the SRH risk and protective factors associated with peers in the context of conflict.

What is known is that peer relationships are present in conflict although the nature and sources of interactions are likely to differ. In the context of armed groups, the development of meaningful relationships may be difficult due to an atmosphere of insecurity, uncertainty and violence. Captives are taught and encouraged to punish other captives, with cases of forced beatings and killings being reported [54]. In these groups, young women can also find themselves one of several ‘co-wives’ to commanders which, due to the ‘protection’ offered by these individuals, can result in competition for affection, resources and power; [55] the manipulation and navigation of these relationships are of great importance. Despite this, examples of the development of positive and long lasting supportive relationships in the context of rebel groups have been reported. Cheney (2007) describes the example of co-wives becoming close friends and confidants as they carry out their duties [54]. In Burman and McKay’s (2007) study of reintegration in the aftermath of the Sierra Leone conflict, three returnee girl mothers were found to be living together [36]. In the context of refugee camps, limited resources such as food may similarly result in competition between peers, whilst interaction with individuals undergoing similar experiences may provide opportunities for support and solidarity.

#### Social interaction

Armed conflict also has been found to impact on social interaction and engagement in the post-conflict period. In Sierra Leone, Bellows and Miguel (2009) found individuals directly affected by violence were more likely than others to be involved in civic participation, such as being members of community and social groups [56]. In Sierra Leone, Denov (2010) found evidence of the creation of informal peer-support structures, where returning girl soldiers sought comfort and encouragement with other conflict affected young people, thereby reducing feelings of isolation [38]. The research procedure itself was found to facilitate this process with research participants forming friendships. Despite limited evidence, insights into the impact of peer relationships on SRH outcomes suggest they may provide a source of support and protection for young women, or may further increase the struggle for securing protective resources.

### Individual level environment

In the context of conflict, the gender, social status and age of young women increase the risk of sexual violence and other poor SRH outcomes [12]. Whilst rape has been used as a weapon for centuries, a new pathology is emerging of ‘rape with extreme violence’ [49]. Such acts performed frequently by soldiers against the women and girls (some very young) aim to cause maximum sexual trauma through injury, mutilation or the transmission of infection [57]. Young people are at greatest risk of abduction by military factions, the vulnerability of girls due to their gender continues after conflict. Whilst focus on children soldiers has been on boys, between 1990 and 2003 in 38 countries girls formed part of forces engaged in armed conflict [58]. It is estimated that 30% of RUF forces in Sierra Leone were made up of girls [59] and, in Northern Uganda, approximately one-third of child soldiers were females [58]. The not uncommon exclusion of girls from disarmament, demobilisation and reintegration processes also means former girl soldiers often have fewer opportunities to develop livelihood strategy skills. For the minority of girls who do get included, the male dominated compositions of these programmes - combined with severe overcrowding and lack of security – often means they are at risk of rape [58].

#### Victims or agents

Haeri and Puechguirbal (2010) warn that, in contexts of conflict, women are generally seen as victims, lacking agency rather than as active individuals who have important characteristics that can make a difference to their circumstances [46]. However, some literature has challenged the very notion of victim’s ‘passivity’, highlighting that even in contexts of captivity, where if recognised, it is possible to evidence every victim’s agency and resistance, to some extent. This literature reframes young women from that of passive victim to one of active agent, able to draw upon personal strengths and resilience to develop strategies which maximise survival chances, including a potential strategy of ‘passivity’.

In the sexual setting of military groups in Northern Uganda and Sierra Leone, McKay and Mazurana (2004) document how girls use their sexuality, sometimes encouraged by their families, to enhance their chances of survival [58]. They give examples in Northern Uganda where young women seek to ‘marry’ or to become pregnant by high commanders due to the associated privileges, such as exceptions from hard labour. In the Revolutionary United Front, ‘family units’ form the basis of organisation with resources being allocated to ‘household heads’. Those that do not belong to a family must scavenge for survival. Girls can use sex and ‘marriage’ to bargain themselves into units and to gain access to food, water and other material goods (also found by Muhwezi et al., 2011; Burman & McKay, 2007) [28, 36].

However, the distinction between expressions of agency and coercion is not always easy to make, as in the case when sexual favours are sought from girls by humanitarian workers in refugee camps in Liberia, Guinea and Sierra Leone in exchange for vital aid supplies, highlighting the inherently exploitive nature of relationships that young women are exposed to in contexts of conflict [43]. The constrained agency and resilience in the actions of young women can have serious consequences for their SRH, especially in relation to motherhood. Yet, while such strategies put young women at risk of pregnancy, forced transaction sex and future exclusion in post-conflict communities, [23, 36] in the short term it can mean their survival. Recognition that early marriage and early sex may have been a strategic decision during war due to limited choices is key for post-conflict strategies which seek to respond to the long-term consequences of this decision.

## Discussion

**Fig. 2:**
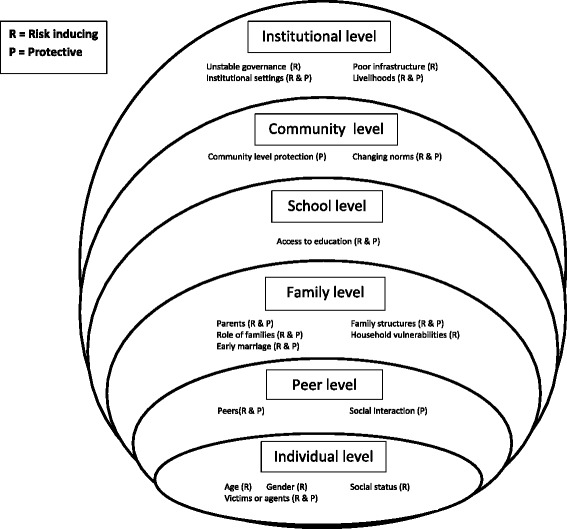
Literature informed ecological framework for risk and protective factors associated with poor sexual and reproductive health outcomes in contexts of armed conflict

The literature highlights the complex nature of how armed conflict impacts the different environments which increase or reduce the risk of poor SRH outcomes for young women – see Fig. 2 for an illustrated version which populates the ecological framework with the risk and protective factors identified in the literature that are associated with poor sexual and reproductive health outcomes in contexts of armed conflict. While there are some similarities with risk and protective factors identified by Blum and Mmari, 2005, it is clear that general models of SRH need reconceptualising in contexts of armed conflict [1]. Diversity in patterns of armed conflict, even within a single country, brings into question presumed protection offered by factors such as proximity to family, access to health professionals or school attendance [60]. While risk factors such as ‘alcohol use’ or ‘knowing where to buy condoms’ or ‘perceived risk of contracting HIV’, for example, were not discussed in the literature reviewed, it can be assumed that the nature of these factors will be impacted by the context of armed conflict. Whilst the literature shows that conflict changes the ecological positions of young people, a lack of consensus exists around the protective nature of some of the factors discussed above. This raises questions of how we understand processes of risk and protection (i.e. how it came to be that certain choices were made/certain outcomes occurred). Knowledge of risk and protection has evolved separately, yet viewing them as distinct entities is unhelpful because of the often complex presence of both risk and protective factors which impact on one another. The factors and environments raised above may act to both expose to, and/or increase, or to protect from risk depending on the individual and context [61, 62]. This is well illustrated by the context of family relationships discussed earlier, which have been shown to be protective against sexual violence at times, while at other times put young women at increased risk of sexual violence through forced or early marriage, or transactional sex. In addition, factors which prevent some poor outcomes like sexual violence, may at the same time increase risk of other poor outcomes like early marriage and early childbearing. Simple binaries need to be challenged, accepting the concepts’ inherent complexities both in relationship to each other and in resilient outcomes. Acceptance that there is no formulaic risk/protection pattern (i.e. a static set of ‘risk’ and ‘protective’ factors which are distinct and categorised) that can be applied for young women in every conflict-affected community is a starting point. This reflects not just the fact that post conflict settings are ‘different’, but rather that the concepts of risk and protection are - by their very nature - dynamic, fluid and contextual.

Rather than focusing on a static list of risk and protective factors, what appear to be important in these contexts are the ‘processes’ of protection - the role of ‘trade-offs’ and the perceived ‘loss and gains’ of actions and choices, the prioritisation of risks in understanding protection strategies, and the ‘price of protection’. The literature clearly shows that there is often a ‘cost’ to securing protection, for example, entering marriage at an early age to ensure security which is often shortly followed by a risky early pregnancy, resulting in the prioritisation of some risks over others. In contexts of conflict, for example, physical safety of oneself or family may be prioritised over immediate or longer-term sexual risks or social exclusion following early motherhood. Starvation or death comes today, whereas the consequences of sexual risks may seem distant [36]. Highlighted is the short and long term nature of protective strategies, as well as the ‘price’ of protection. Risks are often multiple and cumulative, exacerbating the impact of each stressor; this can lead to a spiral of overwhelming risk and adversity exposure [63].

When young women have few choices and resources due to the impact of conflict on protective resources previously available from macro or community environments, sexuality remains as one potential resource which they can draw upon [27]. At the highest levels of risk, protection is either non-existent or fails to counteract the ‘*poisonous effects of extreme adversity*’ (p.140) [64]. Conflict renders such concepts as ‘rights’ and ‘dignities of citizenship’ as obsolete or secondary to saving lives and maintaining essential services [20, 65].

Questions are also raised as to the nature of agency and choice, and the extent to which constrained choice is still choice? Is it possible for young women to be regarded as agentic beings while using their sexuality to access food and temporary security? The role of agency is often viewed as essential in securing assets for protection, with issues of power underpinning the ability to succeed or not [9, 66]. Rutter (2001) suggests that key turning points, the opportunities and choices which might be offered, are the most significant factors in determining resilient outcomes [67]. Power and control are seen as defining the parameters of how and to what extent one can adapt to adversity. Others challenge this focus on personal agency, advocating the prioritisation of addressing structural oppression and social inequalities [68–70]. Seccombe asks, for example, *‘Can families be expected to become resilient without significant structural change in society?’* (p.389) [68]. The ecological examination completed above shows that attention needs to be paid to multiple environments and, more importantly, the relationships between them. While young women may be placed at increased risk by institutional level factors in relation to a particular SRH outcome (such as health clinics being destroyed), for example, the additional risks created could be mitigated against (or further increased) at other levels.

Blum and Mmari (2005) conclude that more studies which identify risk and protective factors for young people focus on individual level factors on SRH rather than contextual factors [1]. Although little is known about the structural and contextual factors which protect young women against poor SRH outcomes even in contexts of relative peace, [1] it is known that these are significantly affected by conflict. Drawing protection from resources at a structural level may therefore not be an option. Young women may have to rely on personal or family assets which serve to protect and increase personal agency while at the same time also increase vulnerability to SRH risks. Indeed, Petchesky (2008) argues for the need to reconnect ‘bodies’ to new communities in times of insecurity [65]. Across the literature there was a scattering of examples of women creating new communities of protection while in contexts of insecurity. For example, Petchesky (2008) reports that:

‘*In Darfur, where the traditional gender division of labour famously assigns women and girls the task of roaming to collect firewood, resulting in a very high incidence of rapes and assaults, committees of women leaders have organised “firewood patrols” which have, in turn, become a forum for discussing and resolving common concerns.’ (p. 8)* [65].

Connecting young women to each other and providing opportunities for action appear to be important actions to facilitate the development of grassroots strategies which support safe negotiations of SRH. Providing young women and their families with access to resources they need to protect themselves recognises the important role of both agency and structure in protection. However, it is not the role of ‘health’ or ‘women’s’ professions alone to support these processes. It is important that professionals working in response to a wide range of concerns in conflict recognise the interconnected nature of SRH with livelihoods, education, gender equality and human rights, and the role that other types of intervention can play in facilitating good SRH [32, 57, 65]. Sexuality and SRH interface with all aspects of life, and therefore need a more integrated response. Efforts to protect young girls and support safe SRH practices should be mainstreamed within all responses to conflict, and vulnerable groups identified and supported. Humanitarian responses focus on meeting survival needs but frequently do not address the cause of, or reasons for, vulnerability [26]. If early marriage or transactional sex is used to secure livelihoods or physical protection, for example, then a focus on improving livelihoods and security might have the biggest impact on improving SRH outcomes.

It is clear that conflict breaks down many protective factors across different environments that might have previously been put into place. However, from the literature available it is difficult to confidently account for how some young women manage to safely negotiate positive SRH outcomes. None of the studies documented accounts of young women successfully negotiating SRH that did not involve putting themselves at risk of some poor sexual outcomes through engaging in risky behaviours. Not enough is known about the difficult choices young women make when there are no ‘positive’ (and safe) choices available (choices without the risk of significant costs in the future) in relation to their SRH. There also appears to be little consideration of the potential paths of resilience during conflict for young women and the protective factors which alter the trajectory from exposure to risk to poor outcomes [9]. It is clear that significant risks will be present in these contexts that may not be avoidable, and yet it is not clear what might prevent or ‘buffer’ the impact of such risks upon an individual and SRH outcomes. The role of post-conflict care in mediating or ‘buffering’ the long-term impact of exposure to such risks is therefore critical, although it is not clear whether there are informed strategies for facilitating this.

The protective resources that a community may hold themselves are not always recognised or appreciated and are sometimes unspectacular, but can be found in the daily activities and struggles of people’s lives [71]. Differences between risk and protection are sometimes only subtle, difficult to predict and only identifiable when family life (girls/young people’s lives) are examined in detail [72]. Ungar, taking a social constructionist approach to the resilience concept, emphasises the need to listen to marginalised and silenced voices - rather than just those of the privileged and powerful - so as better to understand localised definitions of resilience, risk and protection [4]. Interpreting and responding to what is heard poses a challenge as it may not fit with western /professional values/ethical or personal beliefs, particularly around ingrained and sanctified notions of rights/oppression. Humanitarian interventions run the risk of unintentionally propagating Western concepts as definitive knowledge and impairing the recovery and rebuilding process post-conflict. Framing young women solely as victims potentially hides or undermines their resilience and resourcefulness, for example [73]. Yet, research is needed which allows a contextual understanding of protective factors which alter the trajectory of risk exposure to poor SRH outcomes for young women affected by conflict [9]. Identifying these processes has potential to support millions of young women around the world to safely negotiate their SRH needs at a time when they may be prioritised by no one else.

## Conclusion

This paper adds to the emerging literature on the SRH of young women affected by armed conflict by considering the impact that conflict can have on risk and protective environments. A literature overview on the risk and protective factors for SRH in armed conflict has formed the basis for this paper, with findings mapped onto an adapted ecological model to present the ways risk and protective factors, and processes, are evidenced promote or undermine young women’s SRH in conflict. Having considered the findings, we have argued the limitations to traditionally recognised static universal models and understandings of risk and protection, proposing that notions of risk and protection must be nuanced and understood as contextually dependent. We have argued the need for developing frameworks that are able to take account of the dynamic fluidity of risk and protection, so that processes and ‘turning points’ to achieving greater SRH for young women can be identified, understood, and promoted. While acknowledging the important role of agency and choice in securing or undermining a young women’s SRH, we have pointed to the need to explore and reconceptualise the complex nature of individual agency set within wider structural influences that may shape or determine their ability to secure good SRH outcomes. We have discussed the dynamic relationship between individuals, their wider environment, and the complex and often contradictory ways in which protective or risk processes may play out within those environmental levels. This highlights limitations of an individualistic approach to understanding and promoting SRH, and supports the need for ecological based approaches to promoting SRH protective environments for young women. This paper offers no easy answers to the challenges of improving SRH outcomes for young women affected by armed conflict; rather, it seeks to ‘shake up’ any taken for granted assumptions on risk and protection by providing insights into their complexity, pointing towards a need for further work. Such further work will need to take into consideration the processes of protection, the prioritisation of risks, risk trade-offs and the price of protection.

## Abbreviations

DRC: Democratic Republic of Congo
IDP: Internally Displaced Persons
SGBV: Sexual and Gender Based Violence
SRH: Sexual and reproductive health
UN: United Nations
UNFPA: United Nations Population Fund
UNHCR: United Nations High Commissioner for Regugees
